# The Mutual Effect of Marital Quality and Parenting Stress on Child and Parent Depressive Symptoms in Families of Children with Oppositional Defiant Disorder

**DOI:** 10.3389/fpsyg.2017.01810

**Published:** 2017-10-20

**Authors:** Xiuyun Lin, Yulin Zhang, Peilian Chi, Wan Ding, Melissa A. Heath, Xiaoyi Fang, Shousen Xu

**Affiliations:** ^1^Institute of Developmental Psychology, Beijing Normal University, Beijing, China; ^2^CAS Key Laboratory of Behavioral Science, Institute of Psychology, Chinese Academy of Sciences, Beijing, China; ^3^Department of Psychology, University of Macau, Macau, China; ^4^Department of Counseling Psychology and Special Education, Brigham Young University, Provo, UT, United States; ^5^School of Kinesiology and Health, Capital University of Physical Education and Sports, Beijing, China

**Keywords:** oppositional defiant disorder, marital quality, parenting stress, parent depressive symptoms, child depressive symptoms

## Abstract

The purpose of the current study was to examine the mutual relationships between dyadic level (i.e., marital quality and parenting stress) and individual level factors (i.e., children and parental depressive symptoms) in families of children with Oppositional Defiant Disorder (ODD). Specifically, we explored whether marital interaction (marital quality) was associated with symptoms of child depression through parent-child interaction (parenting stress) and parent depressive symptoms. We also explored whether parent-child interaction was associated with symptoms of parent depression through marital interaction and child depressive symptoms. This study was conducted with 256 parent-child dyads, consisting of children with ODD and one of each child's parents. Participants were recruited from 14 primary schools located in northern, eastern, and southwestern China. Results revealed that marital quality predicted symptoms of child depression through the parenting stress, but not parent depressive symptoms; and parenting stress predicted symptoms of parent depression through marital quality, but not through child depressive symptoms. Also, parenting stress significantly and directly predicted parent depressive symptoms. We concluded in families of children with ODD, the association of marital interaction and parent-child interaction on both symptoms of parent and child depression highlighted the mutual effects of the couple subsystem and the parent-child subsystem. Furthermore, in regard to parental and child depressive symptoms, implications for intervention are provided.

## Introduction

The family systems theories reveal that family is an integrated and hierarchically organized system (Gottman, 1979; Nichols and Schwartz, 1998; McHale and Sullivan, 2008). In an attempt to make sense of family relationships, Vose (2010) conducted a dynamic assessment of family function and suggested that the family system could be divided into three levels: whole family level, interaction/dyadic level, and individual level. Marital interaction and parent-child interaction are two important dynamics at the dyadic level within family systems which concern family functioning and the mental health of children and parents (Erel and Burman, 1995; Kwok et al., 2015). A growing literature address the profound impact of marital interaction or parent-child interaction on children's developmental outcomes (Cummings and Davies, 2010; Liu and Wang, 2015a). Other studies have revealed the influence of marital interaction and parent-child interaction on parents' mental health (Whisman, 2001; Proulx et al., 2007; Kouros et al., 2008; National Research Council and Institute of Medicine, 2009).

However, less is known about the interplay of marital interaction and parent-child interaction (on a dyadic level) in affecting both parent and their child mental health (on an individual level). Further, few researchers have investigated these pathways in the context of families with ODD children, in which children and parents were at high risk to develop depressive symptoms (Lerner and Spanier, 2013; Lavigne et al., 2014; Wymbs et al., 2017) and always struggled with impaired family interactions (Benson and Kersh, 2011; Munkvold et al., 2011; Lerner and Spanier, 2013). Focusing on children with ODD and their families, the current study would examine the associations among two main dyadic level factors (marital quality and parenting stress) and two individual level factors (parent and children depressive symptoms).

### Oppositional defiant disorder (ODD)

ODD refers to a recurrent and developmentally aberrant pattern of angry/irritable, negative, defiant, disobedient, and hostile behaviors toward authority figures that are associated with functional impairment; and persist for at least 6 months (American Psychiatric Association, 2000, 2013). As one of the most common mental disorders among children, the prevalence of ODD is estimated at around 1 to 11% (American Psychiatric Association, 2013, p. 464). ODD is identified as a combination of behavioral and emotional problems (Matthys et al., 2013) that is associated with an increased propensity for other behavioral disorders and mood disorders (Greene et al., 2002; Mayes et al., 2016). Specifically, for children with ODD, their irritable mood combined with defiant/headstrong behaviors strain and jeopardize interpersonal relationships (Burke et al., 2014; Cohn and Adesman, 2015). Subsequently, these strained relationships tend to further exacerbate emotional problems and depressive symptoms (Lavigne et al., 2014). In fact, empirical research indicates that ODD has high rates of comorbidity with mood disorders, particularly depression (Lavigne et al., 2014). As children's behavioral problems are strongly associated with emotional problems (Izard et al., 2001), in this study we focused on symptoms of depression among children with ODD.

Previous studies have demonstrated that family dysfunction is closely linked to children emotional problems that are related to ODD, such as anger, hostility, depression, and acute stress (Spertus et al., 2003; Spinazzola et al., 2014). Lin et al. (2013) proposed a multilevel family factors model to explain the effects of family on intensity of ODD symptoms. These family factors included factors at the whole family level, such as low socioeconomic status (SES) and an adverse family atmosphere; factors at the dyadic level, such as poor marital, parent-child, and sibling relationships; and factors at the individual level, such as parent mental health problems and child emotional problems.

Among all factors, the devastating effects of, conflictual and hostile marital or parent-child interaction at a dyadic level on child or parent's psychological outcomes have been extensively documented (Whisman, 2001; National Research Council and Institute of Medicine, 2009; Jeong and Chun, 2010; Liu and Wang, 2015a). More specifically, studies have documented that dysfunctional marital interaction or dysfunctional parent-child interaction is associated with higher levels of children's internalizing problems, externalizing problems, and adverse social adjustment (Spertus et al., 2003; Cummings and Davies, 2010; Jeong and Chun, 2010; Liu and Wang, 2015a). Other studies focused on parents' mental health have revealed that low quality of marital interaction or parent-child interaction predicted the parent's low well-being and more depressive symptoms of parents (Whisman, 2001; Proulx et al., 2007; Farmer and Lee, 2011). However, few studies have simultaneously considered multiple interactions (on a dyadic level) involving the couple subsystem and the parent-child subsystem, exploring their interplay in affecting both child and parent psychological outcomes (on an individual level)—particular in families with children identified with ODD.

### Two possible pathways from marital interaction to child depressive symptoms

Previous research that concentrated on familial factors of child development found an association between poor marital quality and child depressive symptoms (Cummings and Davies, 2010). Furthermore, according to Jeong and Chun's (2010) study, low marital quality is negatively associated with almost every domain of child dysfunction and maladjustment, particularly for child depression. Essex et al. (2003) also found that poor marital quality increased the mental health symptoms of children, including depression. Further, parent-child interaction and parental mental health have been proposed to explain the linkage between marital quality and children psychological outcomes (Essex et al., 2003; Kouros et al., 2014). As such, two pathways are proposed on how distress in the couple subsystem is transmitted to the parent-child subsystem.

The first pathway is supported by the *spillover hypothesis* which describes a transference of mood, affect, or behavior from one subsystem to another (Cox et al., 2001). Based on family systems theories, spillover takes place when strain, negative affection, and conflict in the marital dyad expands beyond the parent relationship and spills over into the parent-child dyad (Kouros et al., 2014). The spillover hypothesis explains a potential pathway in which poor marital quality contributes to a diminished quality in the parent-child relationship, which then exacerbates child emotional problems, such as depressive symptoms (Osborne, 2003). In fact, poor parent-child interactions are commonly recognized as detrimental to healthy child development and are strongly correlated with increased child psychopathology (Chang et al., 2004; Lee et al., 2013). Additionally, empirical studies have shown that higher levels of parenting stress are often accompanied with higher levels of child internalizing problems (e.g., child depressive symptoms; Meijssen et al., 2010; Osborne et al., 2012). From a family systems perspective, highly conflictual marital interactions would predict symptoms of child depression because the marital distress transmits beyond the parents' relationship and negatively affects the parent-child interaction.

The second pathway is that marital interaction influenced on child psychological outcomes through parental mental health problems. Supporting the significant interplay between the married couple relationship and the individual parent mental health, one meta-analysis of 26 cross-sectional studies showed significant negative correlations between marital quality and parent depressive symptoms (−0.42 for women and −0.37 for men; Whisman, 2001). Furthermore, parental psychiatric symptoms are considered high risk factors for emotional and behavioral difficulties in children (Williamson et al., 2004; Burstein et al., 2010). In particular, high levels of maternal depression have increased children's emotional and behavioral problems (Goodman and Tully, 2006). Thus, it is reasonable to anticipate that poor marital quality would predict higher levels of parent mental health problems, ultimately reflected in more symptoms and higher levels of child depression.

### Two possible pathways from parent-child interaction to parent depressive symptoms

Previous research focused on parental mental health found that higher parenting stress resulted in lower marital quality and higher levels of parental psychological distress (Lavee et al., 1996; Kwok et al., 2015). For example, research conducted by Farmer and Lee (2011) indicated that parenting stress was directly associated with poor parent-child interactions and maternal mental health problems, such as depression. Also, there are two pathways to explain the effect of parent-child interaction on parental depressive symptoms through marital interaction and child depressive symptoms respectively, which demonstrated how distress transmit from parent-child subsystem to couple subsystem.

The first pathway is that parenting stress exerted influence on parental depressive symptoms through marital quality. A previous study found a strong and negative relationship between parenting stress and marital quality (Gerstein et al., 2009). According to the family system theory, different levels of factors are interrelated (McHale and Sullivan, 2008). For example, as the *spillover hypothesis* proposes, the negative feelings, stress, and emotions arising from strained parent-child interactions can *spill over* and exert an adverse effect on a couple marital relationship and interactions (Kwok and Wong, 2000; Kwok et al., 2015). In particular, under the high parenting stress of raising maladjusted children, parents tend to complain and quarrel with each other, which jeopardizes marital quality (Cheng and Kuo, 2008). Consequently, parents who experience poor marital quality are more likely to experience negative emotions which increases the risk of experiencing depressive symptoms (Liu and Wang, 2015b).

The second pathway is that parenting stress impacted on parental depressive symptoms through child depressive symptoms. Previous studies found that greater parenting stress is often accompanied with parental mental health through higher levels of child internalizing and externalizing problems (Meijssen et al., 2010; Osborne et al., 2012). Specific aspects of children's emotional and behavioral functioning have been shown to consistently contribute to parents' depressive symptoms—this includes difficult temperament, insecure attachment, emotional intensity, dysregulated outrage and aggression, and discord in interpersonal relationships (National Research Council and Institute of Medicine, 2009). Because children with ODD are more likely to respond oppositionally and irritably in parent-child interactions, maternal depression is frequently associated with the emotional symptoms that often accompany ODD (e.g., anxiety and reactive aggression; Biederman et al., 2001; Cannon, 2015). Hence, it is possible that high levels of parenting stress exacerbate depressive symptoms in children with ODD, subsequently eliciting and intensifying parental depressive symptoms.

### Bidirectional interplays between the couple subsystem and parent-child subsystem

According to bidirectional theories, the interplay between the two predominant subsystems within the family—couple subsystem and parent-child subsystem—is typically identified as bidirectional (Crouter and Booth, 2003). This bidirectional interplay occurs in two ways. On the one hand, the marital interaction influences the parent-child interaction. Two hypotheses explain this effect. One is the aforementioned spillover hypothesis, the other is the compensatory hypothesis. The compensatory hypothesis posits that parents may compensate for their unsatisfying marital relationship by spending more time with their children, subsequently developing a stronger, emotionally supportive parent-child relationship (Erel and Burman, 1995). On the other hand, research also supports the effect of the parent-child interaction on the couple marital interaction (Crouter and Booth, 2003), which also highlights the important role of children influence on the married couple relationship.

### The present study

**Figure 1:**
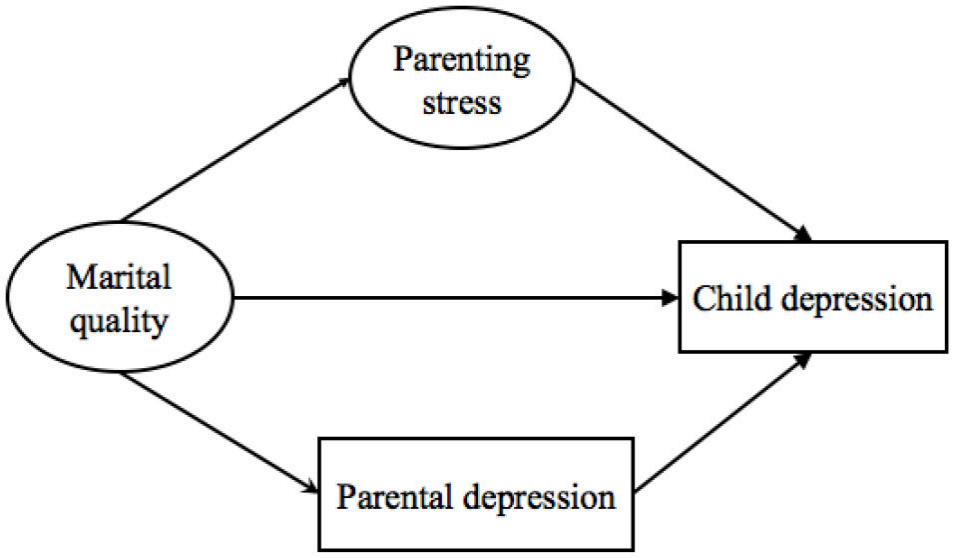
Model 1: The hypothesized pathways from marital quality to child depression.

**Figure 2:**
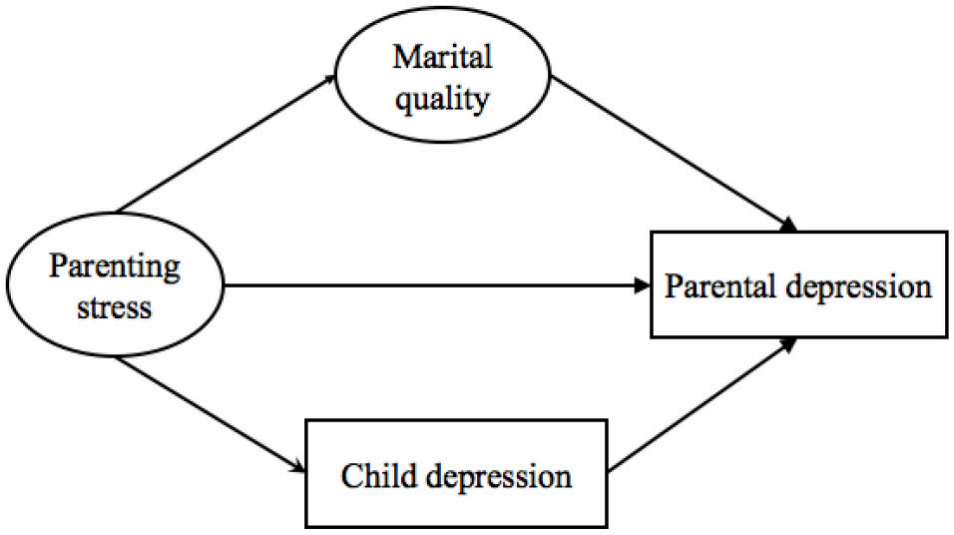
Model 2: The hypothesized pathways from parenting stress to parental depression.

Through certain pathways, marital interactions and parent-child interactions are interplayed, and both marital interactions and parent-child interactions influence child and parent psychological outcomes. Therefore, the present study aimed to examine the mediation model for the pathways from the dyadic level of factors (marital quality and parenting stress) to the individual level of factors (children and parental depressive symptoms). Furthermore, the mutual effects of the couple subsystem and the parent-child subsystem within the family were tested. We hypothesized: (1) Among families with children identified with ODD, the factors at the dyadic level (i.e., marital quality and parenting stress) would be significantly associated with factors at the individual level (i.e., children's depression and parental depression); (2) For the effects of dyadic level factors, marital quality would predict child depressive symptoms through parenting stress, and the pathway from parenting stress to parental depressive symptoms would be mediated by marital quality; (3) For the mutual effect of individual level factors, marital quality would predict symptoms of child depression through parental depressive symptoms, and parenting stress would predict symptoms of parental depression through child depressive symptoms. The hypothesized models are presented in Figures 1, 2.

## Methods

### Description of study's site and sampling

The Institutional Review Board of Beijing Normal University in China approved this study's research protocol and consent procedure. The data in the current study were collected during 2013 and 2014 from 14 elementary schools in Mainland China, including areas in northern (Beijing), eastern (Shandong Province), and southwestern (Yunnan province) China. School psychologists delivered invitation letters—consisting of an introduction of the study and informed consent for class master teachers—to all the participating schools' class master teachers who taught first through fifth grade students. The participating schools' 185 class master teachers were asked to nominate the children who might have ODD symptoms. Teachers were provided with a list of diagnostic criteria taken from the *Diagnostic and Statistical Manual of Mental Disorders*, DSM-IV-TR; (American Psychiatric Association, 2000). Participating teachers nominated 360 children, 4.5% of the 7,966 potential children.

After the initial nomination, two clinical psychologists from Beijing Normal University interviewed the class master teachers to confirm or disconfirm each of the potential ODD diagnoses. Each nominated child's classroom teacher was interviewed with a semi-structured interview that was based on the following DSM-IV-TR diagnostic criteria for ODD (American Psychiatric Association, 2000, p. 102): (a) the child *often* exhibited four or more symptoms of ODD—including losing one's temper, arguing with adults, actively defying and refusing to comply with adult requests or rules, purposefully participating in annoying people, blaming others for his/her mistakes and misbehavior, taking offense by being overly sensitive and easily annoyed by others, displaying angry and resentful feelings, and spitefully seeking vengeance; (b) the identified symptoms were present for at least 6 months or longer; and (c) significant impairments were evident in daily activities and psychosocial functioning.

Based on two clinical psychologists interviewing the class master teachers regarding each of the 360 nominated children, a total of 305 children were identified as displaying the characteristics of ODD (3.8% of the children in the participating schools). In order to obtain parents' and children's consent to participate in the research, invitation letters and consent forms were delivered to parents of the identified 305 children. Of those invited to participate, 282 parent-child dyads signed the informed consent forms. Ultimately the data from 256 parent-child dyads were included in this research study (83.9% participation rate and 3.2% of students in the participating schools). Among the families not attending the study, only 26 families filled in the socio-demographic information. For these 26 families, there were 9 father-child dyads and 17 mother-child dyads, 18 boys and 8 girls. There were no significant difference in child age, (*M*_age_ = 9.04, *SD* = 1.65), parent age (*M*_father age_ = 38.92, *SD* = 4.96; *M*_mother age_ = 36.81, *SD* = 3.87), and SES (60.0% families had a monthly income over 5,000 Chinese Yuan).

The final sample was composed of 256 parent-child dyads (83 father-child dyads and 173 mother-child dyads; *M*_father age_ = 38.45, *SD* = 5.11; *M*_mother age_ = 36.67, *SD* = 4.27) from 156 classrooms. ODD children in the sample consisted of 184 boys (71.9%) and 72 girls (28.1%), ages 6 to 12-years old (*M* = 9.56, *SD* = 1.59). Among these children, 245 children (95.7%) were identified as an only-child in their family. Almost all of the children lived in urban neighborhoods (244, 95.3%). Families were from diverse socioeconomic levels, 56.1% families had a monthly income over 5,000 Chinese Yuan (the average monthly income for Chinese urban families is about 5,485 Chinese Yuan; National Health and Family Planning Commission of the PRC, 2015).

### Survey procedure

After informed consents were returned, children were provided with a packet containing both child questionnaire and parent questionnaire. To prevent the potential harm of being treated differently, children were told that they were randomly selected. Taking ~15 min, children completed their questionnaires in unused school rooms during school hours. To provide assistance when needed, two or three trained researchers stayed in the room as children completed their questionnaire. Each participating child was asked to take a packet containing a parent survey to one of his or her parents. Parents were required to complete their questionnaire at home and return it to the class master teacher within 1 week. Adults were also given the opportunity to discontinue the research at any time. At the end of the survey, each adult participant received a token of appreciation (approximately $8) for their participation.

Additionally, each of the 256 children who met the ODD criteria and their parents were offered opportunities for treatment. Services were available from psychiatrists from Anding Hospital and psychological counselors and family therapists from the Center of Family Study and Therapy at Beijing Normal University.

### Measures

#### Marital quality (parent reported)

Marriage quality was measured using the *Dyadic Adjustment Scale* (DAS; Spanier, 1976). The DAS is a commonly used 32-item self-report inventory assessing the adjustment and satisfaction within marital relationships across four domains: (a) Global Dyadic Satisfaction, (b) Dyadic Cohesion, (c) Dyadic Consensus, and (d) Affectional Expression (Hunsley et al., 2001; Graham et al., 2006). Parents were asked to report the degree to which they agree with the statements or the frequency they behave like the descriptions. Participants assess items, respectively on 2-point scale (*Yes, No*); 5-point scale, ranging from 1 (*Everyday*) to 5 (*None of them*); 6-point scale, ranging from 1 (*Always agree*) to 6 (*Always disagree*); and 7-point scale, ranging from 1 (*Extremely unhappy*) to 7 (*Perfect*). The Dyadic Satisfaction subscale consists of 10 items (e.g., “How often do you and your partner quarrel?”). The Dyadic Cohesion subscale contains five items (e.g., “How often would you and your spouse work together on a project?”). The Dyadic Consensus subscale is composed of 13 items (e.g., “Handing family finances,” “Amount of time spent together,” and “Career decisions”). The Affection Expression subscale includes 4 items, including the following items: “Demonstrations of affection” and “Being too tired for sex.” When interpreting the DAS scores, it is important to note that the 32 DAS items are summed to create a total score ranging from 0 to 151, with higher scores indicating more positive dyadic adjustment and a stronger level of marital quality and satisfaction.

In the current study, the Cronbach's α for the subscales—Dyadic Satisfaction, Dyadic Cohesion, Dyadic Consensus, and Affection Expression—are 0.85, 0.83, 0.93, and 0.61, respectively. The Cronbach's α for the DAS is 0.95. There were no significant differences between fathers' and mothers' reports on the DAS subscales [*t*_subscales(254)_ = 0.15, −0.35, −0.045, 0.07; *t*_total(254)_ = −0.30], *p*s > 0.05.

#### Parenting stress (parent reported)

The 36-item Chinese version of the *Parenting Stress Index–Short Form* (PSI-SF; Reitman et al., 2002) was utilized to assess the mother's or father's perception of parenting stress. Previous research indicates the PSI-SF has proven to be a reliable and valid measurement in Mainland China (Yeh et al., 2001). The PSI-SF has three subscales: Difficult Child, Parent Distress, and Parent–Child Dysfunctional Interaction. Each subscale consists of 12 items. The Difficult Child subscale measures parents' perceptions of their children's characteristics (e.g., “Child cries or fusses more often than other children”). The Parent Distress subscale illustrates the levels of distress that parents feel in regard to their parenting, (e.g., “Feel that I cannot handle things” and “Having a child cause problems with spouse”). The Parent–Child Dysfunctional Interaction subscale describes parents' dissatisfaction when interacting with their children and the degree to which parents find their children unacceptable (e.g., “Child doesn't giggle or laugh much when playing” and “Child smiles at me less than expected”). Parents were asked to report the extent to which they agree with the negative statements on a 5-point scale (1 = *strongly agree* to 5 = *strongly disagree*). The summed scores range from 12 to 60 for Difficult Child, Parent Distress, and Parent–Child Dysfunctional Interaction respectively, with higher score of each subscale indicates a higher level of parenting stress and a lower satisfaction in parent-child relationship. In the current study, the Cronbach's α for these four subscale dimensions were 0.88, 0.84, and 0.87. The Cronbach's α for the PSI-SF is 0.93. There was no significant difference between the fathers' and mothers' reports on each of the PSI-SF subscales [*t*_subscales(254)_ = 1.69, −0.66, 0.23; *t*_total(254)_ = 0.41], *p*s > 0.05.

#### Children depressive symptoms (child reported)

Children's self-reports of depressive symptoms were assessed using the Center for Epidemiological Studies Depression Scale for Children (CES-DC; Fendrich et al., 1990). The scale was composed of 20 items. Sample items include “I was bothered by things that usually don't bother me.” and “My appetite was poor.” Each item is rated on a 4-point scale (0 = not at all, 1 = a little, 2 = some, 3 = a lot). The CES-DC was translated into Chinese in the early 1990s and was validated with various Chinese populations (Wang, 1993). Summed scores were used as measures of children depressive symptoms, with higher scores indicating more severe depressive symptoms. In the current study, the CES-DC Cronbach's α = 0.86.

#### Parental depressive symptoms (parent reported)

Parental depressive symptoms were assessed by the Center for Epidemiologic Studies Depression Scale (CES-D; Radloff, 1977). The CES-D is a self-report scale consisting 20 items, and it covers affective, cognitive, behavioral, and somatic symptoms associated with depressive symptoms. Examples of the items are “I was bothered by things that usually don't bother me.” The CES-D Chinese versions have been used among Chinese samples and have been tested and deemed to be a valid scale (Li and Hicks, 2010). Participants were required to indicate the frequency of the symptoms on a 4-point Likert scale, ranging from 0 (*less than a day*) to 3 (*5–7 days of the past week*). The total score was composed of a sum of all 20 items. Total scores had the potential to range from 0 to 60, with higher scores indicating a higher level of self-reported depressive symptoms. In the current study's data, the Cronbach's α = 0.88. Additionally, there were no significant differences between fathers' and mothers' self-reported levels of depressive symptoms [*t*_total(256)_ = −0.36], *p* > 0.05.

### Data analyses

First, descriptive statistics and intercorrelations among variables at dyadic and individual levels were calculated using SPSS 20.0 (IBM Corporation, 2011). Next, mediating models were examined in Mplus 7.0 within a structural equation modeling (SEM) framework (Muthén and Muthén, 1998, 2015). Bias-corrected 95% confidence intervals for path estimates were generated using bootstrapping with 5,000 iterations (MacKinnon, 2008). Model fit criteria was evaluated using chi-square statistic (χ^2^), the goodness-of-fit index (CFI), the Tucker- Lewis index (TLI), the root mean square error of approximation (RMSEA), and the standardized root mean residual (SRMR). A model is typically accepted as an adequate fit when RMSEA and SRMR < 0.08, CFI and TLI > 0.95 (Hu and Bentler, 1999).

## Results

### Preliminary analyses

The mean and standard deviation of all observed variables, and their intercorrelations are presented in Table 1. The results revealed high interrelation between the variables at dyadic level and individual level. Specifically, there were significant correlations between marital quality, parenting stress, parental depressive symptoms, and child depressive symptoms. Marital quality was negatively associated with child depressive symptoms and parental depressive symptoms, while parenting stress was positively related to child depressive symptoms and parent depressive symptoms.

**Table 1:** Descriptive statistics and correlation matrix.

	***M***	***SD***	**1**	**2**	**3**	**4**	**5**	**6**	**7**	**8**	**9**
1 Satisfaction	45.79	6.37	–								
2 Cohesion	21.63	3.89	0.683^***^	–							
3 Consensus	44.59	11.47	0.557^***^	0.491^***^	–						
4 Affectional expression	12.16	2.63	0.637^***^	0.717^***^	0.387^***^	–					
5 Difficult child	34.08	8.62	−0.323^***^	−0.343^***^	−0.297^***^	−0.263^***^	–				
6 Parent distress	33.64	7.50	−0.305^***^	−0.310^***^	−0.255^***^	−0.296^***^	0.582^***^	–			
7 Dysfunctional interaction	28.16	7.69	−0.339^***^	−0.337^***^	−0.334^***^	−0.271^***^	0.724^***^	0.638^***^	–		
8 Parental depression	10.07	7.61	−0.479^***^	−0.332^***^	−0.337^***^	−0.317^***^	0.420^***^	0.511^***^	0.499^***^	–	
9 Child depression	16.05	10.39	−0.221^***^	−0.182^**^	−0.143^*^	−0.202^**^	0.371^***^	0.257^***^	0.357^***^	0.226^***^	–

* *p < 0.05*,

** *p < 0.01*,

*** *p < 0.001*.

### Two mediator models: mutual interplays between marital quality, parenting stress, parental depressive symptoms, and child depressive symptoms

Two SEM models were examined. There are the pathways from marital quality to child depression through parenting stress and parental depression, and the pathways from parenting stress to parental depression through marital quality and child depression. The fitting coefficients are displayed in Table 2 and indicate good model fits for the two models.

**Table 2:** Fitting coefficients of models.

**Model**	**χ^2^**	***df***	**RMSEA**	**CFI**	**TLI**	**SRMR**
Model 1	70.057	23	0.072	0.950	0.921	0.065
Model 2	33.189	23	0.043	0.989	0.983	0.032

**Figure 3:**
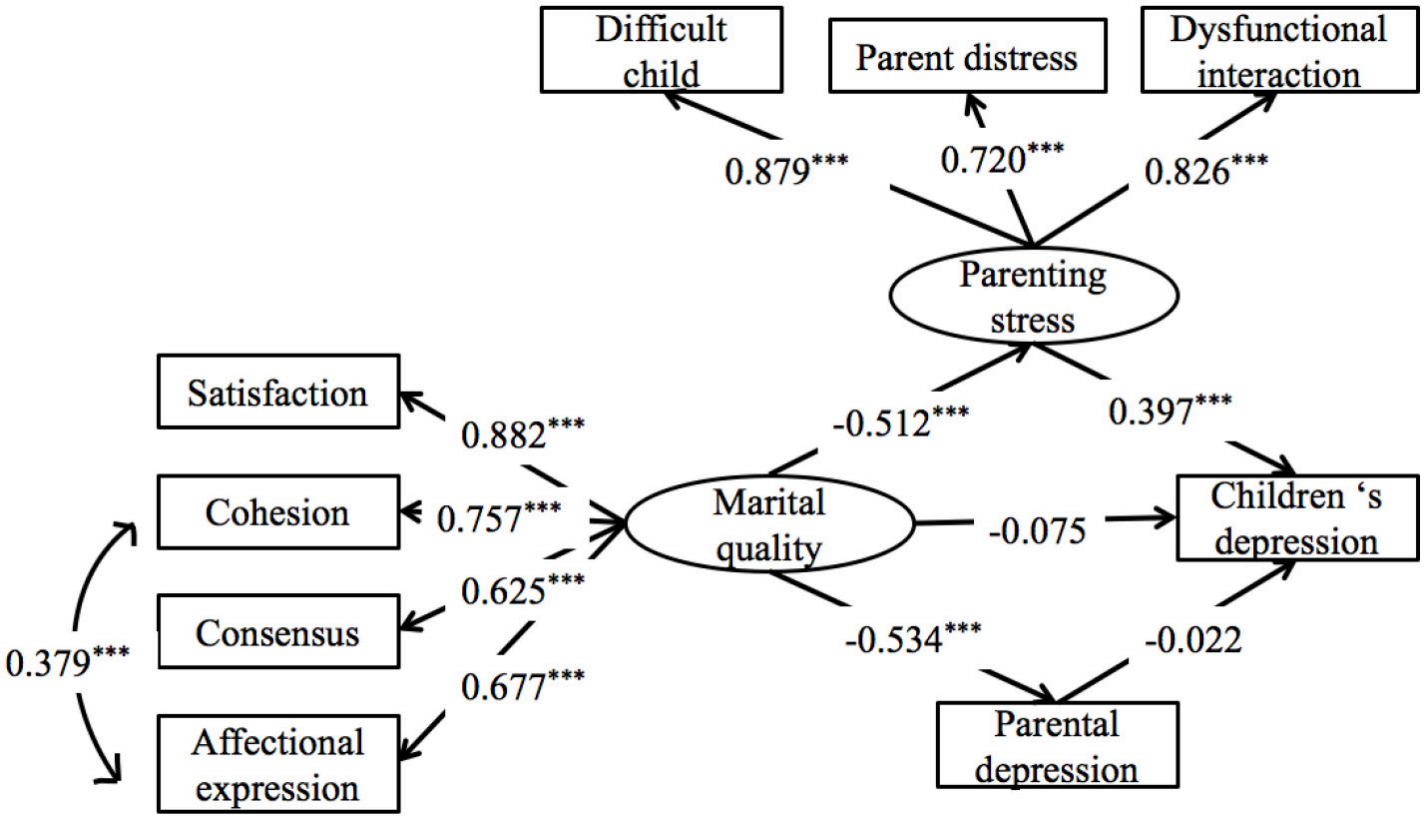
Model 1: The pathways from marital quality to children's depression through parenting stress and parental depression. All the coefficients are standardized estimates. ***p < 0.001.

The results for model 1 were presented in Figure 3 and Table 3. It showed that marital quality had no direct linkage with children depressive symptoms, β = −0.08, *p* = 0.39, 95% CI = [−0.25, 0.09]; but indirectly and negatively associated with children depressive symptoms through parenting stress, β = −0.20, *p* < 0.001, 95% CI = [−0.30, −0.11]. However, the indirect effect of marital quality on child depressive symptoms through parental depressive symptoms was not significant, β = 0.01, *p* = 0.27, 95% CI = [−0.08, 0.10]. Thus, poor marital quality was associated with symptoms of children's depression through stronger parenting stress.

**Table 3:** Indirect, direct, and total effects for models.

**Model**	**Pathways**		***B***	***SE***	***p***	**95%CI**
1	Marriage quality → Parenting stress/Parental depression → Children's depression	Total direct	−0.08	0.09	0.385	[−0.25, 0.09]
		Total indirect	−0.19^***^	0.06	<0.001	[−0.30, −0.08]
		Stress indirect	−0.20^***^	0.05	<0.001	[−0.30, −0.11]
		Depression indirect	0.01	0.05	0.265	[−0.08, 0.10]
2	Parenting stress → Marriage quality/Children's depression → Parental depression	Total direct	0.46^***^	0.07	<0.001	[0.32, 0.60]
		Total indirect	0.12^*^	0.06	0.045	[0.003, 0.24]
		Marital indirect	0.14^**^	0.04	<0.001	[0.06, 0.23]
		Depression indirect	−0.02	0.03	0.484	[−0.09, 0.04]

* *p < 0.05*,

** *p < 0.01*,

*** *p < 0.001*.

**Figure 4:**
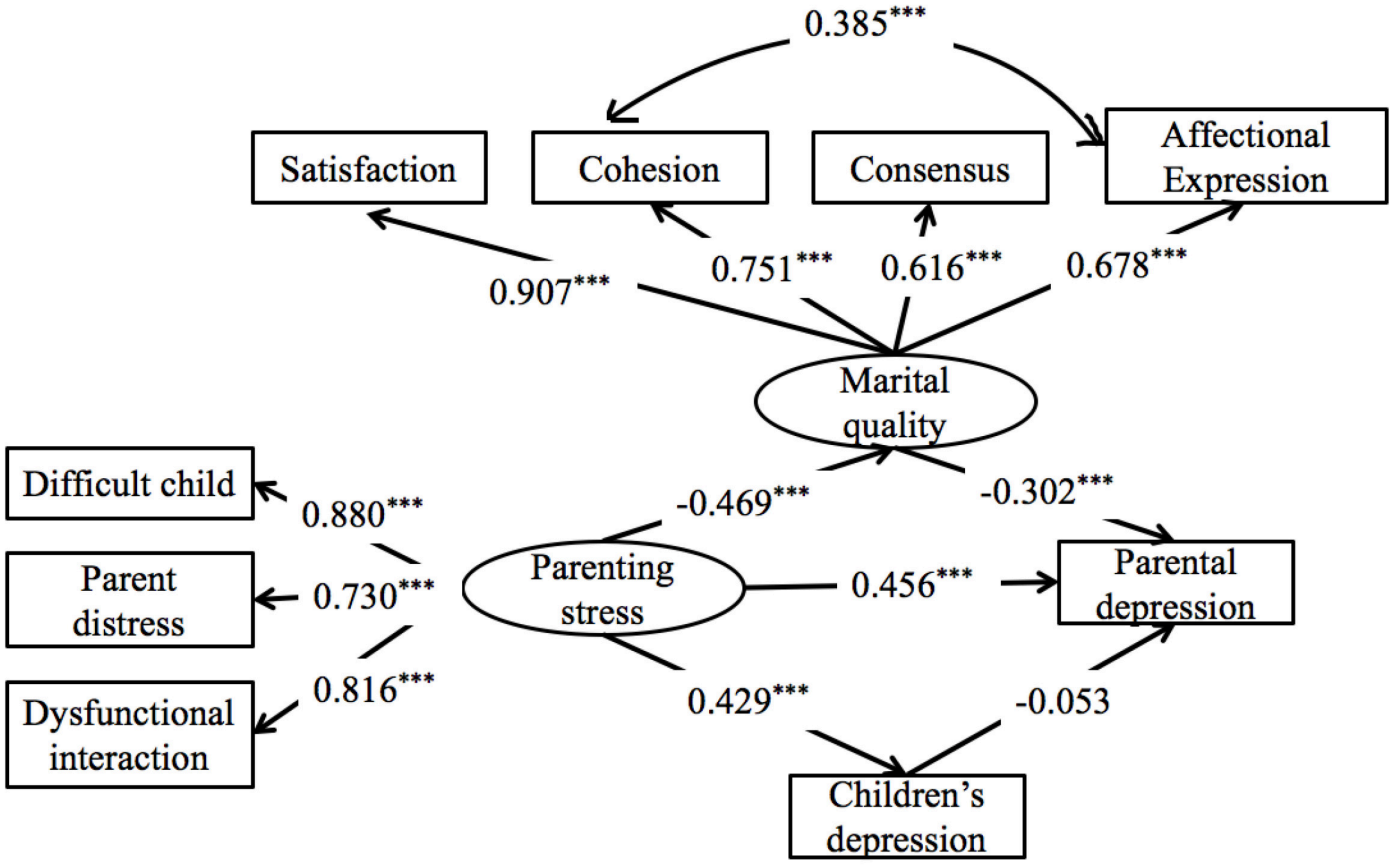
Model 2: The pathways from parenting stress to parental depression through marital quality and child depression. All the coefficients are standardized estimates. ***p < 0.001.

The results for model 2 are presented in Figure 4 and Table 3. It showed that parenting stress predicted parental depressive symptoms directly, β = 0.46, *p* < 0.001, 95% CI = [0.32, 0.60], and indirectly through marital quality, β = 0.14, *p* < 0.001, 95% CI = [0.06, 0.23]. However, the indirect effect of parenting stress on parental depressive symptoms through children depressive symptoms was not significant, β = −0.02, *p* = 0.48, 95% CI = [−0.09, 0.04]. Therefore, low marital quality partially mediated the relationship between parenting stress and the symptoms of parental depression.

## Discussion

This study was conducted in China with 256 parent-child dyads. Based on data collected from parents and their children with ODD, findings in the current study clarified the associations between family factors at the dyadic level and individual level. Specifically, marital quality and parenting stress were significant predictors of parental depressive symptoms, while only parenting stress was a significant predictor of children depressive symptoms. Second, the mediating role of parenting stress in the relationship between marital quality and child depressive symptoms, and the mediating role of marital quality in the relationship between parenting stress and parental depressive symptoms were demonstrated. These findings extended the knowledge regarding the linkages between the dyadic level and the individual level of factors among families of children with ODD. Additionally, this study's findings also contributed empiric bounds of these interrelationships by simultaneously examining the mutual effects of both marital interaction (i.e., marital quality) and parent-child interaction (i.e., parenting stress) on children and parental depressive symptoms. These associations not only supported the spillover effect between the couple subsystem and the parent-child subsystem, but also clearly illustrated pathways for how the distress from one subsystem was transmitted to another. Moreover, one step beyond existing studies, parental depressive symptoms was also involved in the mediation models that were examined in the current study, as well as the inclusion of children depressive symptoms. Having shown the pathways from dyadic level (i.e., marital quality and parenting stress) and individual level factors (i.e., children's depression and parental depression), this finding also contributed to inform the home-based intervention for both children and parents' mental health among families with ODD children.

The present research found that in families of children with ODD, parenting stress positively predicted both parental depressive symptoms and child depressive symptoms directly, while marital quality only had a direct influence on parental depressive symptoms, but not child depressive symptoms. The results were consistent with previous studies, indicating that dyadic level factors were predictive of individual level factors in the family (Whisman, 2001; Lee et al., 2013; Liu and Wang, 2015a). This finding supported the view that proximal factors in the family, such as dysfunctional parenting, affected on children directly (Bronfenbrenner, 2005; Chen et al., 2011). Further, these findings in current study suggested that distal factor (i.e., marital quality) had no effect on the psychological outcomes of children directly and may exert its influence through proximal variables (parenting stress) (Zimet and Jacob, 2001).

The current study also tested the mediation mechanism to evaluate both direct and indirect associations between dyadic-level factors and individual-level factors. Results indicated that there was a mutual effect between marital quality and parenting stress. Moreover, our results indicated that poorer marital quality was significantly associated with children depressive symptoms through parenting stress. This finding supported the *spillover hypothesis* among families included children with ODD (Cox et al., 2001; Kouros et al., 2014). Couples staying in poor quality marriages in which they suffer chronically strained and conflictual marital interactions may become irritable and too emotionally drained to adequately attend to parenting their children, particularly when children have demanding needs (Kouros et al., 2014). These parents may also tend to notice the quarrels and mood-oriented behaviors, which contribute to poor parent-child interactions, such as ignoring and impatiently responding to their children's needs (Osborne, 2003). Moreover, because of their weak emotion regulation skills, children with ODD were at greater risk for experiencing poor parent-child interactions (Cohn and Adesman, 2015) and more likely to encounter difficulties and exhibit dysfunction (Li et al., 2014). Therefore, the low-quality of parent-child interactions that is related to poor marital quality may further aggravate children's psychopathological symptoms. The current study extended the knowledge about effects of distal factors in the family (Grant et al., 2006) by demonstrating that they (e.g., marital quality) exerted their influence on psychological outcomes of children through the proximal factor such as parent-child interaction. Thus, future research and interventions for children with ODD also should concentrate on family-related distal factors, such as marital quality and marital interaction.

The present study also found that low quality of marriage associated with symptoms of parental depression through parenting stress. This finding demonstrated the spillover effect of distress from parent-child subsystem on marital subsystem, extended our knowledge about the family systems theory on the mutual effect between marital interaction and parent-child (Cox and Paley, 1997; Cox et al., 2001). The potential inconsistent and uncooperative parenting behaviors may amplify the predictive power of parenting stress on the deterioration in marital quality (Belsky and Hsieh, 1998). This devastating effect would be even stronger in families of children with ODD because of these children's extreme oppositional and defiant behavioral challenges. Additional support for the linkage between marital quality and parent depressive symptoms, research conducted by Kouros et al. (2008) demonstrated that poor marital quality resulted in more parental depressive symptoms. This process, linkage, and outcome were accomplished over time as a negative family atmosphere undermined the intimacy and cohesion among family members (Davies and Cummings, 1998), which further progressed into a lack of emotional safety and ultimately depressive symptoms in parents (Burke et al., 2000). Thus, negative feelings and pressure spilling over from parenting contribute to poor marital interaction, further resulting in parents experiencing severe depressive symptoms (Kouros et al., 2014; Kwok et al., 2015).

However, in this study, parental depressive symptoms and children depressive symptoms did not strongly predict one another. Inconsistent with findings of previous studies conducted among community samples (Hammen, 2002, 2009), the current study's data demonstrated that in families of children with ODD there were no predictive or strong correlations between these two individual-level factors. There are two possible explanations for this finding. First, children with ODD frequently struggle with associated mood disorders (Hamilton and Armando, 2008). As such, previous research has suggested the possibility that ODD is a common antecedent condition of depression and that the negative affective dimension of ODD is closely associated with depressive symptoms (Burke and Loeber, 2010; Burke et al., 2010). Thus, for children with ODD, their depression may stem from the symptoms of ODD and therefore may be less directly influenced by parental depressive symptoms. Second, it is likely that the present study clarified the limited direct association between children depressive symptoms and parental depressive symptoms by considering the role of related factors at the dyadic level. In this study, when the effects of marital quality and parenting stress were controlled, the depressive symptomatology of parents and children were not predictive of one another. These results suggest the mediating role of interactive factors between parents' and children's individual-level factors, and highlight the vital importance of dyadic-level marital interaction and parent-child interaction, specifically in families with children diagnosed with ODD. Accordingly, it is understandable as to why there is mutual interplay between marital quality and parenting stress, but somewhat less intuitive regarding the minimal mutual effect between children depressive symptoms and parental depressive symptoms.

### Limitations and recommendations for future research

When considering this study's findings, several limitations should be noted. First, the nature of this study's cross-sectional method prevented us from providing a developmental and dynamic model of these relationships across time. Furthermore, our cross-sectional design that utilized questionnaires to assess global marital quality and parenting stress limited us in providing a definitive temporal relationship between these variables, as proposed in the theoretical model (Kouros et al., 2014). Future longitudinal studies will be helpful in refining our understanding of how marital quality and parenting stress influence children depressive symptoms and parental depressive symptoms, and how these interacting processes operate across time.

Second, only one of the parents, not both parents, completed the research measures. Both mother and father's reports of marital and parenting information or multiple methods (e.g., clinical observations) may provide a stronger test of the model. Future research would benefit from collecting both father's and mother's data.

Third, the generalizability of the findings may be challenged because all participants were recruited from families in China who had children with ODD. Further research is needed to examine whether this study's results would also apply to families with children not identified with ODD. Additionally, more research is needed to conduct this type of study in other settings (e.g. western families, families with same-sex parents, etc.), determining the influence of culture and heterogeneous family structures that include a diversity of parenting roles.

Finally the current study only focused on two family factors at the dyadic level (marital quality and parenting stress) and two factors at the individual level (child depression and parent depression). Future studies should explore associations between additional factors (e.g., marital conflict and transgenerational interaction) and individual-level factors (e.g., child aggression and anxiety). Such research would help in providing suggestions for preventing the development of problematic psychopathology in children and adults. Additionally, factors associated with the whole family, such as family cohesion and family climate, were not examined in the current study.

Despite this study's limitations, the present study contributed to a more full understanding of the impact of dyadic-level factors on individual-level factors in families with children diagnosed with ODD. Our findings suggest that when addressing depressive symptoms in children with ODD, interventions and counseling strategies that may prove more effective are those that focus on strengthening parent-child relationships and decreasing parenting stress. Also, our findings may guide future studies by illustrating the importance of considering mutual effects and interactions of the couple subsystem and the parent-child subsystem.

## Author contributions

XL as the first author, wrote the sections of Abstract, Introduction, and part of Discussion. YZ had written the section of Methods and Results. PC gave some suggestions and revised the whole manuscripts. WD had written the part of the Discussion. MH edited and polished the manuscript, particular in language. XF gave some suggestions in revising this manuscript. SX as a corresponding author provided the idea of the whole manuscript and did the data analysis. We worked together to make out this manuscript.

### Conflict of interest statement

The authors declare that the research was conducted in the absence of any commercial or financial relationships that could be construed as a potential conflict of interest.

## References

[B1] American Psychiatric Association (2000). Diagnostic and Statistical Manual of Mental Disorders, 4th Edn. Washington, DC: American Psychiatric Association.

[B2] American Psychiatric Association (2013). Diagnostic and Statistical Manual of Mental Disorders, 5th Edn. Arlington, VA: American Psychiatric Association.

[B3] Belsky J. Hsieh K.-H. (1998). Patterns of martial change during the early childhood years: parent personality, coparenting, and division-of-labor correlates. J. Fam. Psychol. 12, 511–528. 10.1037/0893-3200.12.4.511

[B4] Benson P. R. Kersh J. (2011). Marital quality and psychological adjustment among mothers of children with ASD: cross- sectional and longitudinal relationships. J. Autism Dev. Disord. 41, 1675–1685. 10.1007/s10803-011-1198-9 21347614

[B5] Biederman J. Rosenbaum J. F. Faraone S. V. Hirshfeld-Becker D. R. Friedman D. (2001). Patterns of psychopathology and dysfunction in high-risk children of parents with panic disorder and major depression. Am. J. Psychiatry 158, 49–57. 10.1176/appi.ajp.158.1.49 11136633

[B6] Bronfenbrenner U. (2005). Making human beings human: bioecological perspectives on human development. Sage. Thousand Oaks, CA: Sage Publications.

[B7] Burke J. D. Hipwell A. E. Loeber R. (2010). Dimensions of oppositional defiant disorder as predictors of depression and conduct disorder in preadolescent girls. J. Am. Acad. Child Adolesc. Psychiatry 49, 484–492. 10.1097/00004583-201005000-00009 20431468 PMC2880833

[B8] Burke J. D. Loeber R. (2010). Oppositional defiant disorder and the explanation of the comorbidity between behavioral disorders and depression. Clin. Psychol. Sci. Prac. 17, 319–326. 10.1111/j.1468-2850.2010.01223.x

[B9] Burke J. D. Loeber R. Birmaher B. (2000). Oppositional defiant disorder and conduct disorder: a review of the past 10 years, Part II. J. Am. Acad. Child Adolesc. Psychiatry 41, 1468–1484. 10.1097/00004583-200012000-00007 12410070

[B10] Burke J. D. Rowe R. Boylan K. (2014). Functional outcomes of child and adolescent oppositional defiant disorder symptoms in young adult men. J. Child Psychol. Psychiatry 55, 264–272. 10.1111/jcpp.12150 24117754 PMC3944082

[B11] Burstein M. Ginsburg G. S. Tein J. Y. (2010). Parental anxiety and child symptomatology: an examination of additive and interactive effects of parent psychopathology. J. Abnorm. Child Psychol. 38, 897–909. 10.1007/s10802-010-9415-0 20432062 PMC3362924

[B12] Cannon M. A. (2015). The Relationship among Attention Deficit/Hyperactivity Disorder (ADHD) Subtypes, Oppositional Defiant Disorder (ODD), and Parenting Stress (Order No. AAI3630823). Available online at: http://nsuworks.nova.edu/cps_stuetd/16. from PsycINFO (1685831298; 2015-99100-588).

[B13] Chang L. Lansford J. E. Schwartz D. Farver J. M. (2004). Marital quality, maternal depressed affect, harsh parenting, and child externalising in Hong Kong Chinese families. Int. J. Behav. Devel. 28, 311–318. 10.1080/01650250344000523

[B14] Chen L. Luo X. Wei Z. Guan B. (2011). Parenting styles, parenting locus of control and family function of children with oppositional defiant disorder. Chin. J. Clin. Psychol, 19, 209–211. 10.16128/j.cnki.1005-3611.2011.02.038

[B15] Cheng S. Kuo L. (2008). Marital satisfaction and parent-child triangulation. Bull. Educ. Psychol. 40, 220–238.

[B16] Cohn A. M. Adesman A. (2015). Oppositional defiant disorder and conduct disorder, in Attention-Deficit Hyperactivity Disorder in Adults and Children, eds Adler L. A. Spencer T. J. Wilens T. E. (New York, NY: Cambridge University Press), 139–149.

[B17] Cox M. J. Paley B. (1997). Families as systems. Ann. Rev. Psychol. 48, 243–267. 10.1146/annurev.psych.48.1.243 9046561

[B18] Cox M. J. Paley B. Harter K. (2001). Interparental conflict and parent-child relationships, in Interparental Conflict and Child Development: Theory, Research, and Application, eds Grych J. H. Fincham F. D. (New York, NY: Cambridge University Press), 249–272.

[B19] Crouter A. C. Booth A. (Eds.). (2003). Children's Influence on Family Dynamics: The Neglected Side of Family Relationships. Mahwah, NJ; Lawrence Erlbaum Associates.

[B20] Cummings E. M. Davies P. T. (2010). Marital Conflict and Children: an Emotional Security Perspective. New York, NY: Guilford Press.

[B21] Davies P. T. Cummings E. M. (1998). Exploring children's emotional security as a mediator of the link between marital relations and child adjustment. Child Dev. 69, 124–139. 9499562

[B22] Erel O. Burman B. (1995). Interrelatedness of marital relations and parent-child relations: a meta-analytic review. Psychol. Bull. 118, 108–132. 10.1037/0033-2909.118.1.108 7644602

[B23] Essex M. J. Klein M. H. Cho E. Kraemer H. C. (2003). Exposure to maternal depression and marital conflict: gender differences in children's later mental health symptoms. J. Am. Acad. Child Adolesc. Psychiatry 42, 728–737. 10.1097/01.CHI.0000046849.56865.1D 12921481

[B24] Farmer A. Y. Lee S. K. (2011). The effects of parenting stress, perceived mastery, and maternal depression on parent-child interaction. J. Soc. Serv. Res. 37, 516–525. 10.1080/01488376.2011.607367

[B25] Fendrich M. Weissman M. M. Warner V. (1990). Screening for depressive disorder in children and adolescents: validating the center for epidemiologic studees depression scale for children. Am. J. Epidemiol. 131, 538–551. 10.1093/oxfordjournals.aje.a115529 2301363

[B26] Gerstein E. D. Crnic K. A. Blacher J. Baker B. L. (2009). Resilience and the course of daily parenting stress in families of young children with intellectual disabilities. J. Intellect. Disabil. Res. 53, 981–997. 10.1111/j.1365-2788.2009.01220.x 19874449 PMC2796238

[B27] Goodman S. H. Tully E. (2006). Depression in women who are mothers: an integrative model of risk for the development of psychopathology in their sons and daughters, in Women and Depression: A Handbook for the Social, Behavioral, and Biomedical Sciences, eds Keyes C. L. M. Goodman S. H. (New York, NY: Cambridge University Press), 241–280.

[B28] Gottman J. M. (1979). Marital interaction: Experimental investigations. New York, NY: Academic Press.

[B29] Graham J. M. Liu Y. J. Jeziorski J. L. (2006). The dyadic adjustment scale: a reliablity generalization meta-analysis. J. Marriage Fam. 68, 701–717. 10.1111/j.1741-3737.2006.00284.x

[B30] Grant K. E. Compas B. E. Thurm A. E. McMahon S. D. Gipson P. Y. Campbell A. J.. (2006). Stressors and child and adolescent psychopathology: evidence of moderating and mediating effects. Clin. Psychol. Rev. 26, 257–283. 10.1016/j.cpr.2005.06.011 16364522

[B31] Greene R. W. Biederman J. Zerwas S. Monuteaux M. C. Goring J. C. Faraone S. V. (2002). Psychiatric comorbidity, family dysfunction, and social impairment in referred youth with oppositional defiant disorder. Am. J. Psychiatry 159, 1214–1224. 10.1176/appi.ajp.159.7.1214 12091202

[B32] Hamilton S. S. Armando J. (2008). Oppositional defiant disorder. Am. Fam. Phys. 78, 861–866. 18841736

[B33] Hammen C. (2002). Context of stress in families of children with depressed parents, in Children of Depressed Parents: Mechanisms of Risk and Implications for Treatment, eds Goodman S. H. Gotlib I. H. (Washington, DC: American Psychological Association), 175–199.

[B34] Hammen C. L. (2009). Children of depressed parents, in Handbook of Depression, eds Gotlib I. H. Hammen C. L. (New York, NY: Guilford Press), 275–297.

[B35] Hu L. Bentler P. M. (1999). Cutoff criteria for fit indexes in covariance structure analysis: conventional criteria versus new alternatives. Struct. Equat. Model. 6, 1–55. 10.1080/10705519909540118

[B36] Hunsley J. Best M. Lefebvre M. Vito D. (2001). The seven-item short form of the dyadic adjustment scale: Further evidence for construct validity. Am. J. Fam. Ther. 29, 325–335. 10.1080/01926180126501

[B37] IBM Corporation (2011). IBM SPSS Statistics for Windows, version 20.0. Armonk, NY: IBM Corporation.

[B38] Izard C. Fine S. Schultz D. Mostow A. Ackerman B. Youngstrom E. (2001). Emotion knowledge as a predictor of social behavior and academic competence in children at risk. Psychol. Sci. 12, 18–23. 10.1111/1467-9280.00304 11294223

[B39] Jeong Y. J. Chun Y. J. (2010). The pathways from parents' marital quality to adolescents' school adjustment in South Korea. J. Fam. Issues 31, 1604–1621. 10.1177/0192513X10368269

[B40] Kouros C. D. Papp L. M. Cummings E. M. (2008). Interrelations and moderators of longitudinal links between marital satisfaction and depressive symptoms among couples in established relationships. J. Fam. Psychol. 22, 667–677. 10.1037/0893-3200.22.5.667 18855503

[B41] Kouros C. D. Papp L. M. Goeke-Morey M. Cummings E. M. (2014). Spillover between marital quality and parent-child relationship quality: parental depressive symptoms as moderators. J. Fam. Psychol. 28, 315–325. 10.1037/a0036804 24821519 PMC4543369

[B42] Kwok S. Y. C. L. Cheng L. Chow B. W. Y. Ling C. C. Y. (2015). The spillover effect of parenting on marital satisfaction among Chinese mothers. J. Child Fam. Stud. 24, 772–783. 10.1007/s10826-013-9888-x

[B43] Kwok S. Y. C. L. Wong D. F. K. (2000). Mental health of parents with young children in Hong Kong: the roles of parenting stress and parenting self-efficacy. Child Fam. Soc. Work 5, 57–65. 10.1046/j.1365-2206.2000.00138.x

[B44] Lavee Y. Sharlin S. Katz R. (1996). The effect of parenting stress on marital quality: an integrated mother-father model. J. Fam. Issues 17, 114–135. 10.1177/019251396017001007

[B45] Lavigne J. V. Gouze K. R. Bryant F. B. Hopkins J. (2014). Dimensions of oppositional defiant disorder in young children: Heterotypic continuity with anxiety and depression. J. Abnorm. Child Psychol. 42, 937–951. 10.1007/s10802-014-9853-1 24497230 PMC4090253

[B46] Lee P. C. Lin K. Robson D. Yang H. J. Chen V. C. Niew W. I. (2013). Parent–child interaction of mothers with depression and their children with ADHD. Res. Dev. Disabil. 34, 656–668. 10.1016/j.ridd.2012.09.009 23123879

[B47] Lerner R. M. Spanier G. B. (Eds.). (2013). Child Influences on Marital and Family Interaction: A Life-Span Perspective. New York, NY: Academic Press

[B48] Li W. Li L. Lin X. Liu W. Yang P. Yao J. (2014). Parent-child relationship, teacher-child relationship and peer relationship in children with oppositional defiant disorder. Chin. J. Clin. Psychol. 22, 428–432.

[B49] Li Z. Hicks M. H. (2010). The CES-D in Chinese American women: Construct validity, diagnostic validity for major depression, and cultural response bias. Psychiatry Res. 175, 227–232. 10.1016/j.psychres.2009.03.007 20006386

[B50] Lin X. Li W. Li Y. Zhao Y. Shen J. Fang X. (2013). The family factors and family intervention program for children who have oppositional defiant disorder. Adv. Psychol. Sci. 21, 1983–1995. 10.3724/SP.J.1042.2013.01983

[B51] Liu L. Wang M. (2015a). Parenting stress and children's problem behavior in China: The mediating role of parental psychological aggression. J. Fam. Psychol. 29, 20–28. 10.1037/fam0000047 25438090

[B52] Liu L. Wang M. (2015b). Parenting stress and harsh discipline in China: the moderating roles of marital satisfaction and parent gender. Child Abuse Neglect 43, 73–82. 10.1016/j.chiabu.2015.01.014 25676108

[B53] MacKinnon D. P. (2008). Introduction to Statistical Mediation Analysis. New York, NY: Taylor & Francis.

[B54] Matthys W. Vanderschuren L. J. Schutter D. J. (2013). The neurobiology of oppositional defiant disorder and conduct disorder: Altered functioning in three mental domains. Dev. Psychopathol. 25, 193–207. 10.1017/S0954579412000272 22800761

[B55] Mayes S. D. Waxmonsky J. D. Calhoun S. L. Bixler E. O. (2016). Disruptive mood dysregulation disorder symptoms and association with oppositional defiant and other disorders in a general population child sample. J. Child Adolesc. Psychopharmacol. 26, 101–106. 10.1089/cap.2015.0074 26745442 PMC4800381

[B56] McHale J. P. Sullivan M. J. (2008). Family systems, in Handbook of Clinical Psychology, Vol. 2, eds Hersen M. Gross A. M. (New York, NY: John Wiley & Sons), 192–226.

[B57] Meijssen D. E. Wolf M. J. Koldweijin K. Van-Wassenaer A. G. Kok J. H. Van-Baar A. L. (2010). Parenting stress in mothers after very preterm birth and the effect of the infant behavioral assessment and intervention program. Child Care Health Dev. 37, 195–202. 10.1111/j.1365-2214.2010.01119.x 20645992

[B58] Munkvold L. H. Lundervold A. J. Manger T. (2011). Oppositional defiant disorder—Gender differences in co-occurring symptoms of mental health problems in a general population of children. J. Abnorm. Child Psychol. 39, 577–587. 10.1007/s10802-011-9486-6 21243524

[B59] Muthén L. K. Muthén B. O. (1998). Mplus: Statistical Analyses with Latent Variables. Mplus User's Guide, 3. Los Angeles, CA: Muthén & Muthén.

[B60] Muthén L. K. Muthén B. O. (2015). Mplus: Statistical Analyses with Latent Variables. Mplus User's guide, 7th Edn. Los Angeles, CA: Muthén and Muthén

[B61] National Research Council and Institute of Medicine (2009). Depression in Parents, Parenting, and Children: Opportunities to Improve Identification, Treatment, and Prevention. Washington, DC: The National Academies Press Available online at: https://www.ncbi.nlm.nih.gov/books/NBK215117/pdf/Bookshelf_NBK215117.pdf 25009931

[B62] Nichols M. P. Schwartz R. C. (1998). Family Therapy: Concepts and Methods, 4th Edn. Needham Heights, MA: Allyn & Bacon.

[B63] Osborne C. Berger L. M. Magnuson K. (2012). Family structure transitions and changes in maternal resources and well-being. Demography 49, 23–47. 10.1007/s13524-011-0080-x 22215507 PMC3570825

[B64] Osborne L. N. (2003). Neuropsychological and Psychosocial Predictors of Children's Depression (Order No. 3086150). Available from ProQuest Dissertations and Theses A&I: The Sciences and Engineering Collection. (305331119).

[B65] Proulx C. M. Helms H. M. Buehler C. (2007). Marital quality and personal well-being: A meta-analysis. J. Marriage Fam. 69, 576–593. 10.1111/j.1741-3737.2007.00393.x

[B66] Radloff L. S. (1977). The CES-D scale: a self-report depression scale for research in the general population. Appl. Psychol. Meas. 1, 385–401. 10.1177/014662167700100306

[B67] Reitman D. Currier R. O. Stickle T. R. (2002). A critical evaluation of the Parenting Stress Index-Short Form (PSI-SF) in a head start population. J. Clin. Child Adolesc. Psychol. 31, 384–392. 10.1207/S15374424JCCP3103_10 12149976

[B68] Spanier G. B. (1976). Measuring dyadic adjustment: new scales for assessing the quality of marriage and similar dyads. J. Marriage Fam. 38, 15–28. 10.2307/350547

[B69] Spertus I. L. Yehuda R. Wong C. M. Halligan S. Seremetis S. V. (2003). Childhood emotional abuse and neglect as predictors of psychological and physical symptoms in women presenting to a primary care practice. Child Abuse Neglect 27, 1247–1258. 10.1016/j.chiabu.2003.05.001 14637300

[B70] Spinazzola J. Hodgdon H. Liang L. J. Ford J. D. Layne C. M. Pynoos R. (2014). Unseen wounds: The contribution of psychological maltreatment to child and adolescent mental health and risk outcomes. Psychol. Trauma 6:S18 10.1037/a0037766

[B71] Vose J. J. C. (2010). Assessment of Family Functioning at Multiple Levels: An Exploratory Investigation of the Dynamic Assessment of Family Functioning Inventory-Demonstrated Under Clinical Conditions (DAFFI-DUCC): How Despicable is the DAFFI-DUCC? (Order No. AAI3434191). DAI-B 72/02, Dissertation Abstracts International Alfred University, New York, NY.

[B72] Wang X. (1993). Rating Scales for Mental Health (Chinese Journal of Mental Health Supplement). Beijing: Chinese Association of Mental Health.

[B73] Whisman M. A. (2001). The association between depression and marital dissatisfaction, in Marital and Family Processes in Depression: A Scientific Foundation for Clinical Practice, ed Beach S. R. H. (Washington, DC: American Psychological Association), 3–24.

[B74] Williamson D. E. Birmaher B. Axelson D. A. Ryan N. D. Dahl R. E. (2004). First episode of depression in children at low and high familial risk for depression. J. Am. Acad. Child Adolesc. Psychiatry 43, 291–297. 10.1097/00004583-200403000-00010 15076262

[B75] Wymbs B. T. Dawson A. E. Egan T. E. Sacchetti G. M. Tams S. T. Wymbs F. A. (2017). ADHD and depression symptoms in parent couples predict response to child ADHD and ODD behavior. J. Abnorm. Child Psychol. 45, 471–484. 10.1007/s10802-016-0220-2 27796690

[B76] Yeh C. H. Chen M. L. Li W. Chuang H. L. (2001). The Chinese version of the parenting stress index: a psychometric study. Acta Paediatrica 90, 1470–1477. 10.1111/j.1651-2227.2001.tb01615.x 11853348

[B77] Zimet D. M. Jacob T. (2001). Influences of marital conflict on child adjustment: review of theory and research. Clin. Child Fam. Psychol. Rev. 4, 319–335. 10.1023/A:1013595304718 11837462

